# Amputation at the Shoulder Joint

**Published:** 1873-10

**Authors:** J. F. Miner


BUFFALO

Medical and Surgical Journal.

VOL XIII. OCTOBER, 1873. No. 3.

### Original Communications.

:o:

ART. I.—*Amputation at the Shoulder Joint.* Remarks and Cases. By J. F. Miner, M. D.

The rule in Surgery, which seems to be fully established, that the nearer the body the greater the risk in amputations, suggests the propriety in hip and shoulder joint operations of considering carefully any measures of procedure calculated to diminish this risk. Prof. McGraw, of Detroit, suggested the capital idea in hip joint amputations of removing the bone from the acetabulum, when practicable, and dividing the soft parts at a greater distance from the body, thus avoiding the division of so great amount of tissue, and really removing the amputation to the junction of the upper and middle third, or even lower than this point in some cases. I had practiced upon this idea in injuries of the shoulder joint, but had not considered the importance of the principles involved until reading his paper, when the value of the suggestion was better appreciated.

In injuries of the bone and soft parts about the shoulder, it is not very unusual to be able to remove the shattered bone from the joint, and divide lower down upon the arm the lacerated soft parts, thus retaining, after recovery, some portion of the soft parts of the arm, which in the usual method of amputation at the shoulder joint were wholly sacrificed. These boneless stumps are of no great value in the motions or adjustments of artificial arms, but they are not altogether useless; they maintain the contour of the shoulder, and above all, it is believed that oftentimes the risks attending the operative procedure are greatly lessened, and the operation, in controlling hemorrhage and tying the vessels, much simplified. The results of some recent cases have impressed me with the conviction that when the system is greatly reduced by loss of blood or from effects of long continued disease, the value of this procedure cannot be over-estimated.

A patient entered the General Hospital, at my request, for the purpose of exsection of a part or the whole of the humerus, as found necessary. Being bloodless and suffering from profuse purulent discharge, it was hoped that increase of strength and flesh might be obtained by a few weeks delay of the operation. Disappointed in this, it was soon apparent that he must lose his arm or life, or probably both, the disorganization of the arm being complete, having become a suppurating mass from within five or six inches of the joint throughout. The idea of making the usual operation of amputation at the shoulder joint was scarcely feasible, as our patient was hardly alive and could not be expected to bear much operative interference. The diseased bone was divided near the middle with a chain saw and the upper fragment carefully detached from the soft parts and glenoid cavity, and removed. The vessels were now easily controllable by grasping through the soft parts, and at a distance of about five inches from the joint the soft parts were divided, the vessels tied and parts approximated and retained by adhesive plaster, and warm water dressings applied. To the surprise of us all, the patient did not seem to suffer from the operation, no blood was lost, and but little living tissue was divided. He made a good recovery, gained rapidly in flesh and strength, and left the hospital in three months fully recovered.

The second case of this character was from railroad injury. The attempt to save the arm having failed, Dr. Green, of Buffalo, invited me to amputate at the shoulder joint. Mortification had left a line of demarkation four or five inches from the joint. The bone was shattered and now partially separated from the soft parts. With but little hope of saving the life of this patient, we yet removed the bone to the joint, divided what of tissue remained alive at about five inches distant from the body, laid the parts gently together, retaining them with plaster, and applying warm water dressings. This patient recovered without an unpleasant symptom after the operation, from a condition of depression and bloodlessness, which according to my observation is generally fatal.

My third case is a recent one, and more strongly impressed me with the advantages of removing the bone first and then dividing soft parts at as great a distance from the body as circumstances will permit. A young man had an arm lacerated and completely destroyed by railroad accident. The soft parts were torn to within about four inches of the body, the bone broken to near the joint, the fragments loose and pointing into the flesh in all directions. The patient, suffering from shock and loss of blood, was so nearly pulseless that it seemed scarcely proper to make any interference; but after delaying for the effects of stimulants a little, the head of the bone was carefully disarticulated from the glenoid cavity and the brachial artery ligated. After this, the remaining soft parts were divided at a distance of about four inches from the body, and the parts loosely approximated with a suture or two and adhesive strips. The patient rallied and gradually recovered. This process consists in simplifying the operation as much as possible, and in this I believe consists the advantage of the procedure.

